# New prognosis indexes specific to Barcelona Clinic Liver Cancer stage B hepatocellular carcinoma combining functional hepatic reserve and tumor burden

**DOI:** 10.1093/oncolo/oyag392

**Published:** 2026-10-05

**Authors:** Koji Fujita, Rie Yano, Mai Nakahara, Kyoko Oura, Hironobu Suto, Asahiro Morishita, Takashi Himoto, Keiichi Okano, Hideki Kobara

**Affiliations:** Department of Gastroenterology and Neurology, Faculty of Medicine, Kagawa University, Takamatsu 760-8521, Japan; Department of Gastroenterology and Neurology, Faculty of Medicine, Kagawa University, Takamatsu 760-8521, Japan; Department of Gastroenterology and Neurology, Faculty of Medicine, Kagawa University, Takamatsu 760-8521, Japan; Department of Gastroenterology and Neurology, Faculty of Medicine, Kagawa University, Takamatsu 760-8521, Japan; Department of Gastroenterological Surgery, Faculty of Medicine, Kagawa University, Takamatsu 760-8521, Japan; Department of Gastroenterology and Neurology, Faculty of Medicine, Kagawa University, Takamatsu 760-8521, Japan; Department of Medical Technology, Kagawa Prefectural University of Health Sciences, Takamatsu 761-0123, Japan; Department of Gastroenterological Surgery, Faculty of Medicine, Kagawa University, Takamatsu 760-8521, Japan; Department of Gastroenterology and Neurology, Faculty of Medicine, Kagawa University, Takamatsu 760-8521, Japan

**Keywords:** ALBI grade, Barcelona Clinic Liver Cancer stage, hepatocellular carcinoma, TNM classification

## Abstract

**Background:**

Patients with Barcelona Clinic Liver Cancer stage B (BCLC-B) hepatocellular carcinoma (HCC) are characterized by heterogeneity in functional hepatic reserve and tumor burden. Conventional prognosis indexes are not specific to this stage. This study aimed to establish novel prognosis indexes that integrated functional hepatic reserve and tumor burden in patients with BCLC-B HCC.

**Methods:**

A total of 1843 patients with BCLC-B HCC were retrospectively enrolled from 4 hospitals. The primary endpoint was overall survival. Tumor burden was quantified as the biproduct or sum of the maximum diameter and the number of tumor nodules that is referred to as mMDN and mUS. Functional hepatic reserve was described using the albumin-bilirubin (ALBI) score.

**Results:**

Multivariate analysis demonstrated that functional hepatic reserve and tumor burden were significantly associated with overall survival. Novel prognosis indexes, termed mMDN/ALBI and mUS/ALBI, were generated by dividing mMDN and mUS by the absolute value of the ALBI score. The mMDN/ALBI and mUS/ALBI models demonstrated superior prognostic stratification of overall survival compared with the ALBI and Tokyo scores, as reflected by higher log-rank trend chi-square statistics and C-index values. Time-dependent ROC and decision curve analyses further supported their superior predictive performance and clinical utility. mMDN/ALBI ≥ 8.2 indicated poor overall survival with a median survival time of 12 to 14 months whether functional hepatic reserve was preserved or not. mUS/ALBI ≥ 5.6 also suggested the median survival time of 9 to 12 months.

**Conclusion:**

The novel prognosis indexes stratified overall survival more clearly compared to the ALBI score and Tokyo score. These indexes enable more accurate prognosis prediction for patients with BCLC-B HCC.

Implications for practice The prognosis for patients with hepatocellular carcinoma is determined by tumor burden and functional hepatic reserve. In particular, for BCLC Stage B hepatocellular carcinoma, the combination of tumor burden and functional hepatic reserve results in a wide range of clinical manifestations among patients. The prognostic indexes newly developed in this study consist of simple ratios, with tumor burden as the numerator and liver functional reserve as the denominator. By predicting the prognosis of patients with BCLC Stage B hepatocellular carcinoma with greater accuracy than existing prognostic indexes, these new indicators can aid in determining treatment strategies.

## Introduction

Barcelona Clinic Liver Cancer (BCLC) stage B occupies a unique position in the pathogenesis and therapeutic strategy of hepatocellular carcinoma (HCC).^1–4^ Patients with BCLC stage B exhibit wide variability in tumor burden and heterogeneity in the functional hepatic reserve of the underlying liver.^5^^,^^6^ Regarding tumor burden, BCLC stage B is further divided into 4 stages, B1 to B4.^7^ Alternatively, it can be classified based on the therapeutic response to transarterial chemoembolization (TACE), as either “within” or “beyond” the up-to-seven criteria.^8–10^ In addition, functional hepatic reserve in BCLC stage B is stratified into at least 2 categories; Child–Pugh classification A or B, or based on the modified albumin–bilirubin (ALBI) grade, as 1 to 2a or 2b to 3.^11^

Conventional prognosis indexes, including the Child–Pugh classification and the ALBI grade, primarily focus on functional hepatic reserve.^12^ Integrating both tumor burden and functional hepatic reserve, some prognosis indexes have been proposed from Japan including the Japan Investigated Staging score (JIS score), the modified ALBI-T (mALBI-T) score, and Tokyo score.^11^^,^^13^^,^^14^ However, these integrated prognosis indexes are not specific to BCLC stage B, for these indexes were generated based on patients with BCLC stage 0 to C or D. The current study aimed to establish prognosis indexes for patients with BCLC stage B HCC.

## Methods

### Ethics

This study was conducted in accordance with the ethical guidelines of the Declaration of Helsinki^15^ and was approved by the institutional review board (IRB) of Kagawa University, Faculty of Medicine (serial number: 2025-115). The requirement for informed consent was waived by the IRB, as the clinical data used for analyses were publicly accessible.

### Study design

This retrospective cohort study included patients from multiple hospitals. The primary endpoint was overall survival. For analyses, the cohort was divided into 4 quartiles (Q1, Q2, Q3, and Q4) in ascending order of any variable of interest.

The sample size of the cohort was limited owing to the retrospective nature of the study. The study was conducted in accordance with the Strengthening the Reporting of Observational Studies in Epidemiology statement.^16^

### Patients

Clinical data were obtained from the Sun Yat-sen University Cancer Center (SYSUCC) database.^17^ The data of 3 cohorts were downloaded from the DRYAD data repository (https://datadryad.org/stash/dataset/doi:10.5061/dryad.pd44k8r).

The derivation cohort consisted of 979 consecutive patients with newly diagnosed HCC at SYSUCC between January 2007 and May 2012. The internal testing cohort included 627 patients with HCC treated at SYSUCC between June 2012 and December 2015. The multicenter testing cohort comprised 414 patients from 3 hospitals; the Second Hospital of Guangzhou Medical University, and the Third and Fifth Affiliated Hospital of Sun Yat-sen University.

According to the original report,^17^ all patients (1) met the inclusion criteria for clinical evaluation of BCLC stage B; (2) had complete follow-up data, including blood test and imaging; and (3) had no history of other malignancies.

In addition to the inclusion criteria described in the original report,^17^ the exclusion criteria specific to the current study were applied: unclear tumor location, presence of ascites or insufficient documentaion regarding the presence of ascites, distant metastasis, and missing or erroneous baseline data on serum albumin or total bilirubin levels.

All patients in the original 3 cohorts presented with 2 or more nodules at the diagnosis or initial treatment for HCC. According to the original description,^17^ TACE was the first-line treatment in 91.7% of patients. In case of complete remission, follow-up was conducted every 3 months during the first 2 years; and extended to every 6 months thereafter.

### Clinical data

The following clinical data were included in the analyses: age, sex, serum albumin concentration, total bilirubin concentration, prothrombin time (PT), alpha-fetoprotein (AFP) concentration, number of intrahepatic nodules, largest tumor diameter, observation period, and outcomes (death or censored). While both PT (s) and PT (%) were recorded in the derivation cohort, PT (%) values were not recorded in the internal testing or multicenter testing cohorts. PT (%) in two cohorts was estimated based on the data of the derivation cohort; PT = 70% was 14.1 s and PT = 40% was 18.5 s .

The ALBI score was calculated as follows: log_10_ total bilirubin (µmol/L) × 0.66   albumin (g/L) × (−0.085).^12^ The modified ALBI (mALBI) grade was defined as grade 1 (ALBI score < −2.60), grade 2a (ALBI score ≥ −2.60 and  < −2.27); grade 2b (ALBI score ≥ −2.27 and  < −1.39); and grade 3 (ALBI score ≥ −1.39).^11^

The JIS score was calculated as the sum of the Child–Pugh classification and HCC staging defined by the Japan Society of Hepatology (JSH).^13^^,^^18^ Numbers 0, 1, and 2 were assigned to Child–Pugh A, B, and C, respectively. Similarly, Numbers 0, 1, 2, and 3 were assigned to HCC stage 1, 2, 3, and 4, respectively.

The mALBI-T score was defined as the sum of the modified ALBI (mALBI) grade and HCC staging defined by JSH.^11^^,^^18^ Numbers 0, 1, 2, and 3 were assigned to the mALBI grade 1, 2a, 2b, and 3, respectively. Numbers 0, 1, 2, and 3 were assigned to HCC stage 1, 2, 3, and 4, respectively.

Tokyo score was calculated based on serum albumin, total bilirubin, the maximum tumor diameter, and number of tumor nodules.^14^ Each parameter was divided into 3 parts and assigned by numerical variables 0, 1, or 2. Tokyo score then counted the sum of 4 parameters ranging 0 to 8.

### Tumor burden indexes

Tumor burden was assessed based on the maximum tumor diameter (mm) and the number of tumor nodules (1, 2, 3, or 4). Cases with more than 4 tumor nodules were assigned a count of 4.

Tumor burden indexes were developed using the multiplication or sum of the maximum tumor diameter (mm) and number of tumor nodules. According to the idea of multiplying the maximum tumor diameter by the number of tumor nodules (MDN), a modified index was developed and termed modified MDN (mMDN), calculated as: mMDN = maximum tumor diameter (mm)/10 × number of tumor nodules.^19^ Similarly, drawing on the concept of the “up-to-seven criteria,” a second index was established by sum ming the maximum tumor diameter and the number of tumor nodules named “modified Up-to-seven” (mUS): mUS = rounded maximum tumor diameter (mm)/10   number of tumor nodules.^8^^,^^17^

For example, in the multicenter cohort, the patient ID 295 had a maximum tumor diameter of 28 mm and 4 nodules. According to the formulas above, mMDN = 2.8 × 4 = 11.2, and mUS = 3   4 = 7.

### Statistical analyses

Continuous variables are presented as medians and interquartile ranges and were analyzed using the Mann–Whitney *U* test. Categorical variables were analyzed using the chi-square or Fisher’s exact tests. A Cox proportional hazard model was employed to determine the hazard ratios (HRs) of functional hepatic reserve and tumor burden. A trend in overall survival among 3 or 4 sub-groups was evaluated using the log-rank trend test. Survival curves for two groups were compared using the log-rank test. Concordance index and time-dependent ROC analyses were performed to assess the discriminative ability of the indexes, while decision curve analysis was used to evaluate their potential clinical utility. Statistical significance was set at *P* < .05. All analyses were performed using GraphPad Prism version 6 (GraphPad Software, La Jolla, CA, USA), except for the concordance index, time-dependent ROC, and decision curve analyses, which were performed using R version 4.6.0 through RStudio.

## Results

### Patient selection

From a total of 2020 patients, we excluded 144 patients with ascites or distant metastasis, 3 with unclear tumor location, 28 with missing data on serum albumin or total bilirubin levels, 2 with an apparent written mistake on serum albumin or total bilirubin levels. Finally, the cohort included 1843 patients with HCC (Figure S1, see online supplementary material for a color version of this figure).

### Baseline characteristics of the patients

Baseline characteristics of the patients are summarized in Table 1. The cohort consisted of relatively young patients, with 75% of all patients younger than 65 years old. Functional hepatic reserve was compensated in approximately 65% of patients, classified as the mALBI grade 1 to 2a. However, the remaining 35% had impaired functional hepatic reserve, with the mALBI grade 2b to 3. According to Child–Pugh classification, 86.9% of patients were class A.

**Table 1.** Baseline characteristics of patients.

	Total	HR	95% CI	*P* value
**Case number**	1843	–	–	–
**Age**	54 (44-62)	1.005	0.999-1.011	.0772
**Male/female**	1262/581	1.185	1.021-1.376	.0254
**Albumin (g/L)**	38.6 (35.1-42.2)	–	–	–
**Total bilirubin (µmol/L)**	17.9 (12.3-26.8)	–	–	–
*Prothrombin time (second, *n* = 1796)	12.1 (11.5-12.9)	–	–	–
**ALBI score**	−2.458 (−2.800 to −2.102)	–	–	–
**ALBI grade (1/2a/2b/3)**	725/462/620/36 (39.3/25.1/33.6/2.0%)	1.118	1.038-1.204	.0032
*Child Pugh score (*n* = 1796)	5 (5-6)	–	–	–
*Child Pugh classification (A/B/C, n = 1796)	1560/232/4 (86.9/12.9/0.2%)	–	–	–
**Number of tumor nodules (2/3/4 or more, ％)**	573/142/1128 (31.1/7.7/61.2%)	1.274	1.181-1.375	<.0001
**Maximum tumor diameter (mm)**	65 (43-94)	1.013	1.011-1.015	<.0001
**TNM classification based on JSH guideline (1/2/3/4, %)**	0/46/1797/0 (0/2.5/97.5/0%)	–	–	–
**TNM classification based on UICC guideline (1/2/3/4, %)**	0/0/1843/0 (0/0/100/0%)	–	–	–
**AFP (ng/mL, *n* = 1737)**	231.7 (16.9-4397)	1.000	1.000-1.000	.0727
*JIS score (1/2/3/4,%, *n* = 1796)	37/1532/223/4 (2.1/85.3/12.4/0.2%)	–	–	–
**mALBI-T (1/2/3/4/5, %)**	20/715/468/604/36 (1.1/38.8/25.4/32.8/2.0%)	–	–	–
**Tokyo score (1-2/3/4/5/6-8, %)**	306/350/560/374/253 (16.6/19.0/30.4/20.3/13.7%)	–	–	–
**Modified MDN**	20 (12-32)	–	–	–
**Modified US**	10 (8-13)	–	–	–
**mMDN/ALBI**	8.202 (4.824-13.86)	–	–	–
**mUS/ALBI**	4.099 (2.975-5.629)	–	–	–
**Observation period (months)**	17 (8-32)	–	–	–
**Censored/death**	897/946	–	–	–

*Prothrombin time (%) data were missing in 47 patients.Abbreviations: ALBI score, the albumin-bilirubin score; HR, hazards ratio; JIS score, the Japan Investigated Score; JSH, Japan Society of Hepatology; mALBI-T, the modified ALBI-T score; MDN, multiplication of maximum tumor diameter and number of tumor nodules; UICC, Union for International Cancer Control; US, Up-to-seven.

In terms of tumor burden, more than 60% of patients had 4 or more HCC nodules. The maximum tumor diameter ranged from 8 mm to 218 mm. While the tumor burden of the cohort showed heterogeneity in terms of maximum diameter and number of tumor nodules, the TNM classification defined by JSH classified 97.5% of patients as stage 3. Similarly, the TNM staging based on UICC assigned all patients into stage 3. In contrast, the novel tumor burden indexes, mMDN and mUS, ranged widely from 3.2 to 87.2, and from 5 to 26, respectively.

Among integrated indexes of functional hepatic reserve and tumor burden, the JIS score classified more than 85% of patients as score 2 (Table 1). Based on the mALBI-T score, more than 95% of patients distributed within score 2 to 4. With respect to Tokyo score, patient numbers with score 1, 2, 6, 7, and 8 were *n* = 86, 220, 187, 58, and 8, respectively. In the current cohort, patients with score 1 and 2, and patients with score 6 to 8, were constructed into stand-alone groups, respectively.

Prothrombin time was not recorded in 47 patients, so Child–Pugh classification/grade and the JIS score were not calculated in the corresponding patients (Table 1).

### Novel prognosis indexes integrating tumor burden with the ALBI score

A multivariate analysis was performed using a Cox proportional hazard ratio model to determine HRs of the mALBI grade and tumor burdens. As shown in Table 1, the advance in the mALBI grade 1 to 3 significantly correlated with poor overall survival (HR = 1.118; 95% confidence interval (CI), 1.038–1.204; *P* = .0032). Both the maximum tumor diameter (HR = 1.013; 95% CI, 1.011–1.015; *P* < .0001) and the number of tumor nodules (HR = 1.274; 95% CI, 1.181–1.375; *P* < .0001) significantly correlated with poor outcomes. Serum AFP was not an independent risk factor for overall survival (HR = 1.000; 95% CI, 1.000–1.000; *P* = .0727). Age did not contribute to overall survival, neither (HR = 1.005; 95% CI, 0.999–1.011; *P* = .0772).

Using absolute values of the ALBI score and tumor burden indexes (mMDN and mUS), 2 prognosis indexes were generated: mMDN/ALBI, which is mMDN divided by the absolute value of the ALBI score; and mUS/ALBI, which is mUS divided by the absolute value of the ALBI score.

### Basic characters of novel prognostic indexes

The correlation between the ALBI score and tumor burden was significant but trivial with r = 0.0767 for mMDN (*P* = .0010), and with r = 0.0642 for mUS (*P* = .0059), as shown in Figure S2A and B (see online supplementary material for a color version of this figure). mMDN/ALBI correlated strongly with mMDN with r = 0.9428 (*P* < .0001); it correlated weakly with the ALBI score with r = 0.3783 (*P* < .0001, Figure S2C and D, see online supplementary material for a color version of this figure). mUS/ALBI also correlated strongly with mUS with the r = 0.8540 (*P* < .0001); and moderately with the ALBI score moderately with r = 0.5402 (*P* < .0001), as presented in Figure S2E and F (see online supplementary material for a color version of this figure).

### Stratification of overall survival using novel prognostic indexes

To compare the prognostic stratification ability of the indexes, the total cohort was divided into Q1, Q2, Q3, and Q4 in ascending order, based on the variable of interest. The log-rank trend test was performed for the ALBI score, mMDN/ALBI and mUS/ALBI, as shown in Figure 1. All 3 indexes significantly stratified the prognosis of the 4 sub-cohorts. Chi-square in mMDN/ALBI was 20 times greater than that in the ALBI score (283.7 vs. 14.1). For mUS/ALBI, the chi-square was also 17 times greater than that in the ALBI score (243.0 vs. 14.1). The HRs for overall survival between Q4 and Q1 were 2 times greater in mMDN/ALBI (HR = 3.958; 95% CI, 3.857–5.704; *P* < .0001) and mUS/ALBI (HR = 3.828, 95% CI, 3.788–5.604, *P* < .0001) compared to that of the ALBI score (HR = 1.515; 95% CI, 1.270–1.817; *P* < .0001).

**Figure 1.**
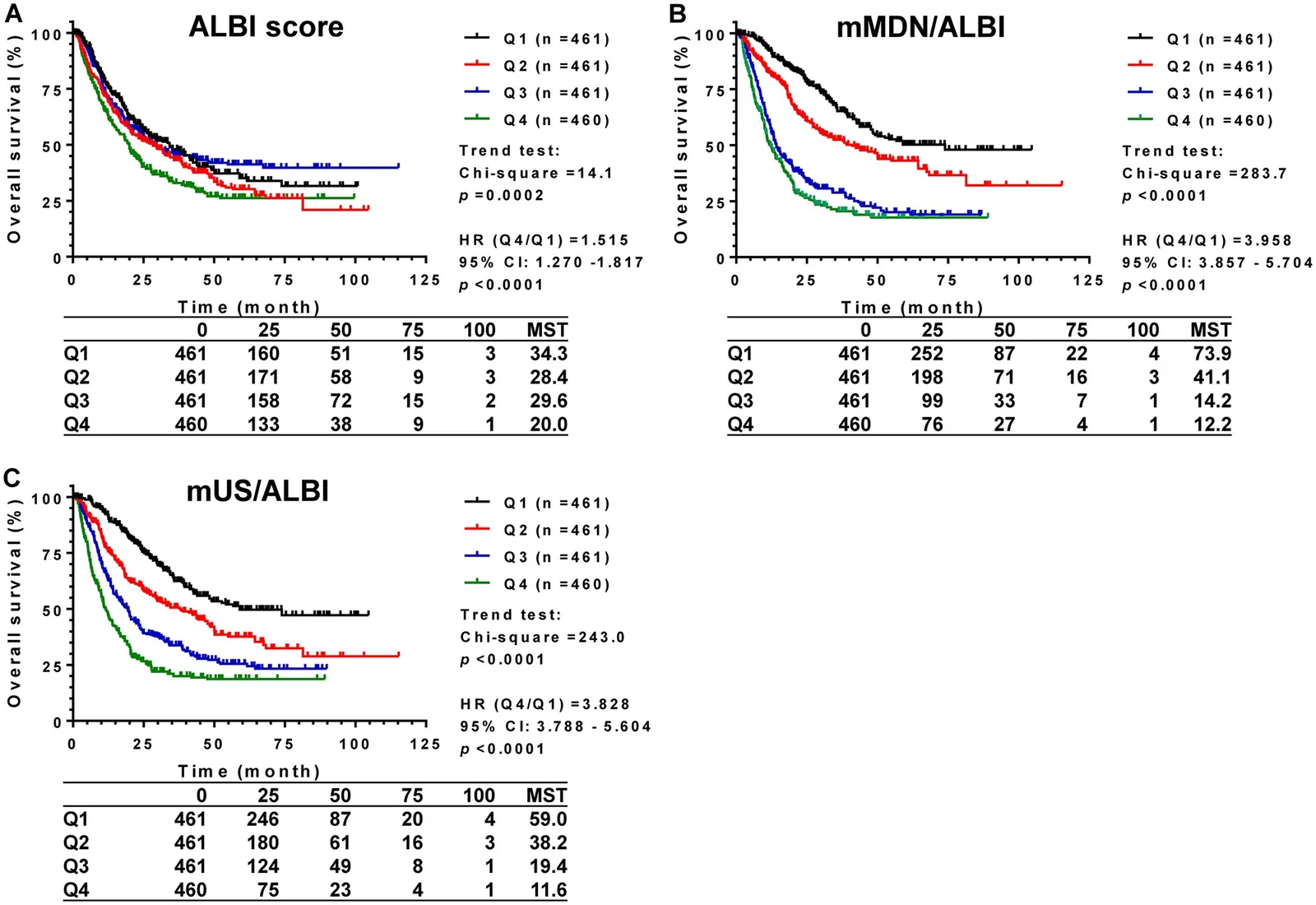
Stratification of overall survival in the total cohort. The total cohort was divided into 4 quartiles (Q1, Q2, Q3, and Q4) based on the ALBI score, mMDN/ALBI, or mUS/ALBI. (A) The ALBI score, (B) mMDN/ALBI, and (C) mUS/ALBI significantly stratified the overall survival (*P* < .0001). The chi-square values of mMDN/ALBI and mUS/ALBI were more than 10 times greater compared to the ALBI score in log-rank trend test. Hazard ratios of Q4 over Q1 were more than 2 times greater in mMDN/ALBI and mUS/ALBI compared to that in the ALBI score. Abbreviations: HR, hazard ratio; MST, median survival time.

### Stratification of overall survival using conventional integrated indexes

Three prognostic indexes integrating functional hepatic reserve and tumor burden were also evaluated for prognosis stratification of the current cohort: the JIS score, the mALBI-T score, and Tokyo score. As shown in Figure 2A and B, most patients were classified as having a JIS score of 2 and 3, and an mALBI-T score of 2, 3, and 4. Compared to mMDN/ALBI and mUS/ALBI, the log-rank trend tests calculated smaller chi-square values for the JIS score and the mALBI-T score: 26.0 for the JIS score and 18.4 for the mALBI-T score.

**Figure 2.**
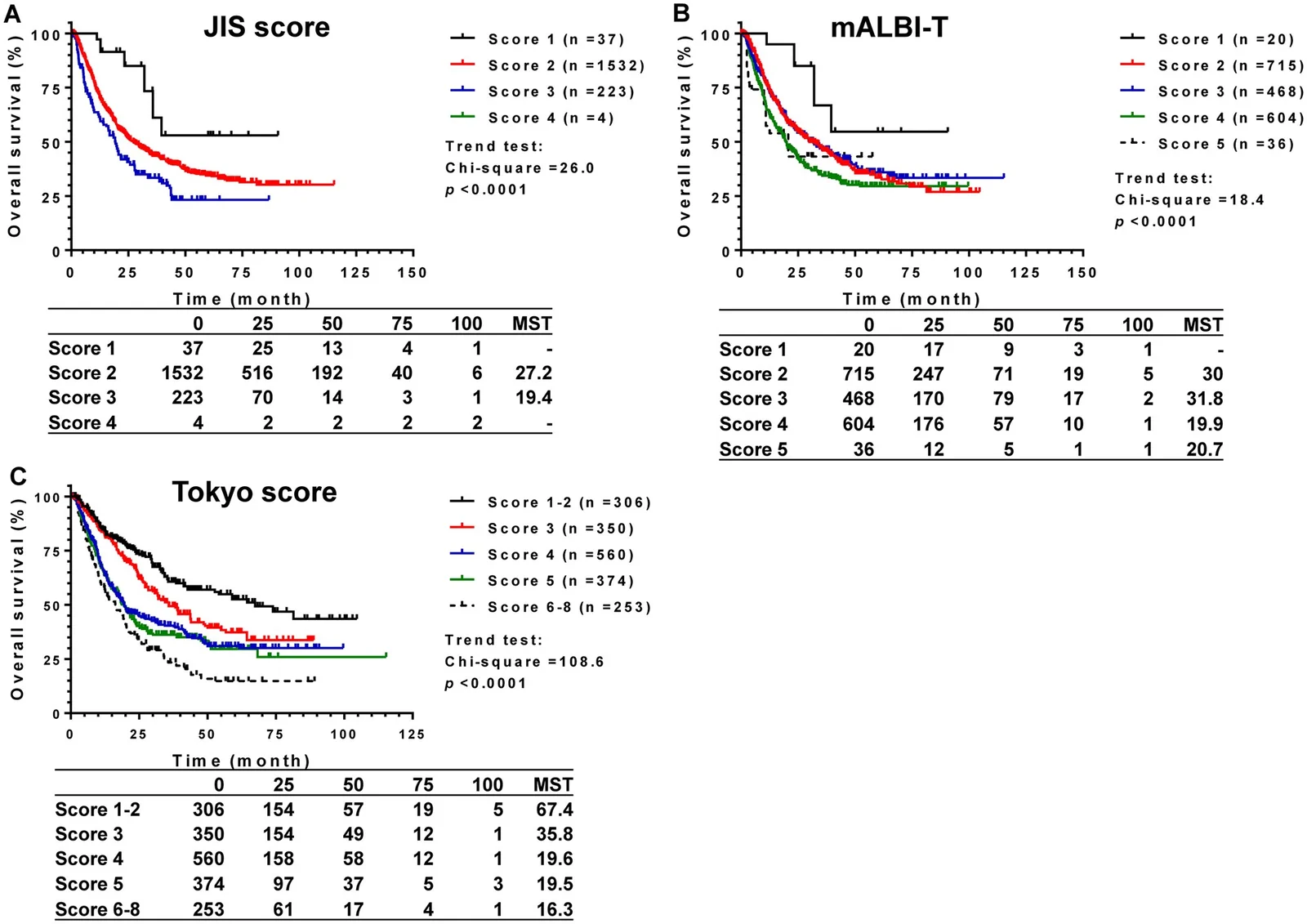
Conventional prognosis indexes integrating functional hepatic reserve and tumor burden. Three conventional prognosis indexes, the JIS score, the mALBI-T score, and Tokyo score were applied on the total cohort. The JIS score was combination of Child–Pugh classification with the TNM classification. The mALBI-T score consisted of the modified ALBI grade and the TNM system. Tokyo score assigned scores = 0, 1, or 2 to each of serum albumin, total bilirubin, the maximum tumor diameter, and the number of tumor nodules. (A) The JIS score classified more than 1500 patients into score 2. Patients less than 3% of the total cohort were assigned into score 1 or score 4. The overall survival of 4 sub-cohorts was significantly stratified with chi-square = 26.0 (*P* < .0001). (B) Based on the mALBI-T score, more than 95% of all patients were evaluated as score 2 to 4. Log-rank trend test significantly differentiated the overall survival with chi-square = 18.4 (*P* < .0001). (C) Tokyo score distinguished the overall survival of the cohort with chi-square = 108.6 (*P* < .0001). Abbreviation: MST, median survival time.

Tokyo score has common property with mMDN/ALBI and mUS/ALBI in that they quantify tumor burden by the maximum tumor diameter and number of tumor nodules. When the current cohort was classified into 5 sub-cohorts (score 1–2, 3, 4, 5, and 6–8), Tokyo score differentiated the overall survival with chi-square = 108.6 (*P* < .0001, Figure 2C).

### Comparison of prognostic performance among indexes

The prognostic stratification ability of each index was evaluated using the concordance index (C-index) and time-dependent receiver operating characteristic (ROC) analysis. Among the evaluated models, mMDN/ALBI demonstrated the highest predictive accuracy, with a C-index of 0.685 (95% CI, 0.668–0.702), followed by mUS/ALBI (0.674 [95% CI, 0.657–0.691]) and the Tokyo score (0.608 [95% CI, 0.589–0.626]), as shown in Table 2. The C-index for the ALBI score was 0.545 (95% CI, 0.525–0.565).

**Table 2.** Comparison of C-index.

	C-index	95% confidence interval
**ALBI score**	0.545	0.525-0.565
**mMDN/ALBI**	0.685	0.668-0.702
**mUS/ALBI**	0.674	0.657-0.691
**JIS score**	0.535	0.522-0.547
**mALBI-T**	0.551	0.532-0.569
**Tokyo score**	0.608	0.589-0.626

Abbreviations: ALBI score, the albumin-bilirubin score; C-index, concordance index; JIS score, the Japan Investigated Score; mALBI-T, the modified ALBI-T score; mMDN, modified multiplication of maximum tumor diameter and number of tumor nodules; mUS, modified Up-to-seven.

Time-dependent ROC analysis further supported these findings. The AUC values for mMDN/ALBI and mUS/ALBI were consistently higher than those of the other indexes throughout the observation period, particularly at 6, 12, 18, and 24 months (Table 3). At 12 months, the AUC (95% CI) values were 0.742 (0.716–0.767) for mMDN/ALBI, 0.730 (0.704–0.756) for mUS/ALBI, 0.631 (0.603–0.659) for the Tokyo score, and 0.560 (0.530–0.591) for the ALBI score. Similar trends were observed at 24 months.

**Table 3.** Time-dependent ROC analysis.

	6 months	12 months	18 months	24 months
**ALBI score**	0.600 (0.560-0.639)	0.560 (0.530-0.591)	0.540 (0.511-0.570)	0.541 (0.511-0.571)
**mMDN/ALBI**	0.750 (0.720-0.780)	0.742 (0.716-0.767)	0.748 (0.723-0.773)	0.747 (0.721-0.773)
**mUS/ALBI**	0.753 (0.722-0.783)	0.730 (0.704-0.756)	0.725 (0.700-0.751)	0.723 (0.696-0.750)
**JIS score**	0.571 (0.542-0.600)	0.539 (0.520-0.558)	0.533 (0.514-0.551)	0.533 (0.514-0.552)
**mALBI-T**	0.603 (0.566-0.640)	0.569 (0.540-0.598)	0.548 (0.520-0.576)	0.551 (0.523-0.579)
**Tokyo score**	0.631 (0.595-0.666)	0.631 (0.603-0.659)	0.637 (0.610-0.664)	0.652 (0.624-0.680)

Data are presented as area under curve (95% confidence interval).

Abbreviations: ALBI score, the albumin-bilirubin score; JIS score, the Japan Investigated Score; mALBI-T, the modified ALBI-T score; mMDN, modified multiplication of maximum tumor diameter and number of tumor nodules; mUS, modified Up-to-seven; ROC, receiver operating characteristic curve.

Decision curve analysis demonstrated differences in the clinical utility of the evaluated indexes (Figure S3, see online supplementary material for a color version of this figure). Within a certain range of thresholds, 2 models based on mMDN/ALBI and mUS/ALBI showed a higher net benefit than the alternative models, as well as the treat-all and treat-none strategies.

Overall, the C-index and time-dependent ROC analyses demonstrated superior discriminative ability, while decision curve analysis indicated greater clinical utility for the mMDN/ALBI and mUS/ALBI models compared with the other scoring systems.

### Prognosis stratification based on tumor burden

Using the median value of mMDN = 20, the total cohort was divided into 2 sub-cohorts. Each of 2 sub-cohorts was also divided into 4 quartiles by quartile values of mMDN/ALBI in each sub-cohorts. In patients with mMDN ≥ 20, mMDN/ALBI stratified the prognosis of 4 sub-cohorts with chi-square = 24.4 (*P* < .0001), as shown in Figure S4A (see online supplementary material for a color version of this figure). The HR between Q4 over Q1 was 1.758 (95% CI, 1.439–2.292; *P* < .0001). In the remaining sub-cohort with mMDN < 20, the prognosis trend was also statistically significant with chi-square = 36.9 (*P* < .0001), as shown in Figure S4B (see online supplementary material for a color version of this figure). The HR between Q1 and Q4 was 2.423 (95% CI, 1.882–3.330; *P* < .0001).

Prognosis prediction using mUS/ALBI was also evaluated under greater and smaller tumor burdens using the median mUS value = 10 (Figure S4C and D, see online supplementary material for a color version of this figure). When patients were selected using mUS ≥ 10, mUS/ALBI significantly differentiated the overall survival of the cohort with chi-square = 24.7 (*P* < .0001). The HR of Q4 over Q1 was 1.596 (95% CI, 1.299–2.031; *P* < .0001). Patients with mUS < 10 also showed similar results in log-rank trend analysis (chi-square = 23.0, *P* < .0001) and the log-rank test of Q4 over Q1 (HR = 2.003; 95% CI, 1.513–2.719; *P* < .0001).

### Prognosis stratification depending on functional hepatic reserve

Prognosis prediction by 2 novel indexes was evaluated based on functional hepatic reserve. Patients were divided into 2 sub-cohort: the mALBI grade 1 or 2a, and grade 2b or 3, based on the cutoff value of ALBI score = −2.27. In this analysis, cutoff values of mMDN/ALBI and mUS/ALBI were determined referring to the quartile values in the total 1843-patient cohort: 4.8, 8.2, and 13.8 for mMDN/ALBI; 2.9, 4.1, and 5.6 for mUS/ALBI (Table 1).

When patients were limited to those with the mALBI grade = 1 or 2a, mMDN/ALBI (chi-square = 182.2, *P* < .0001) and mUS/ALBI (chi-square = 158.8, *P* < .0001) significantly stratified the overall survival of the sub-cohort, as shown in Figure 3A and B. mMDN/ALBI ≥ 8.2 and mUS/ALBI ≥ 5.6 resulted in a poorer prognosis with a median survival time between 9 and 14 months. Tokyo score stratified the prognosis of the sub-cohort with a smaller chi-square = 56.1 (*P* < .0001), compared to that in novel indexes (Figure 3C).

**Figure 3.**
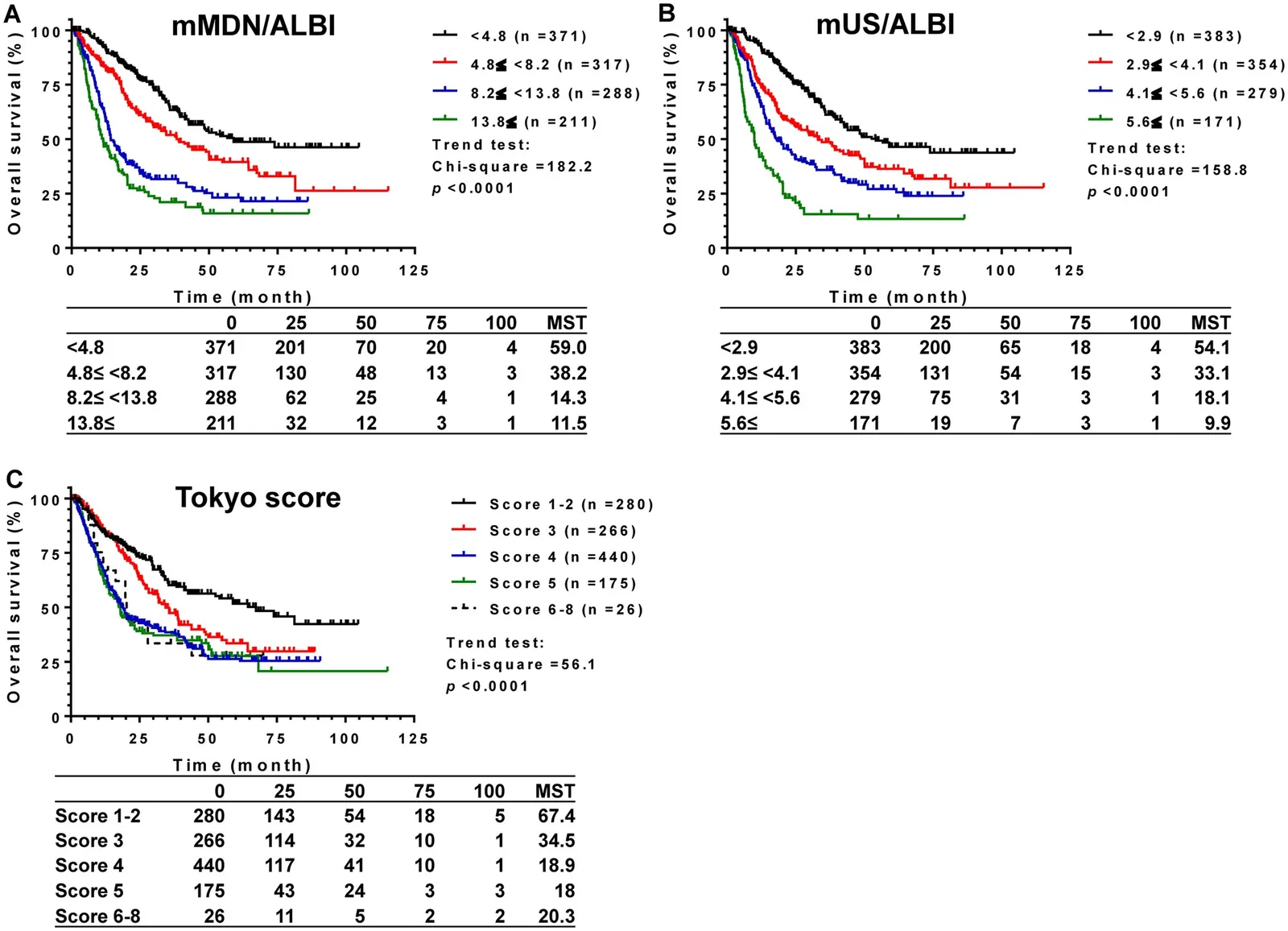
Stratification of overall survival by mMDN/ALBI and mUS/ALBI under the condition of the modified ALBI grade = 1 or 2a. Novel indexes were compared to Tokyo score for prognosis prediction in patients with preserved functional hepatic reserve. Patients with mALBI grade = 1 or 2a were subject to the analysis (*n* = 1187). The cutoff values of mMDN/ALBI (4.8, 8.2, and 13.8) and mUS/ALBI (2.9, 4.1, and 5.6) were selected from the quartile values of them in the total cohort. (A, B) In case of mALBI grade 1 to 2a, mMDN/ALBI and mUS/ALBI significantly differentiated the overall survival of the sub-cohort (chi-square = 182.2, *P* < .0001 for mMDN/ALBI; and chi-square = 158.8, *P* < .0001 for mUS/ALBI). mMDN ≥ 8.2 indicated the median survival time 11 to 14 months. mUS/ALBI≥ 5.6 also resulted in around 10 months of the median survival time. (C) Tokyo score stratified the overall survival with smaller chi-square = 56.1 (*P* < .0001) compared to those of mMDN/ALBI and mUS/ALBI.

In the case of the mALBI grade = 2b or 3, both indexes differentiated the overall survival of the sub-cohort; chi-square = 88.1 for mMDN/ALBI (*P* < .0001); and chi-square = 72.3 for mUS/ALBI (*P* < .0001), as shown in Figure 4A and B. When the mALBI grade = 2b or 3 was assumed, mMDN/ALBI ≥ 8.2 and mUS/ALBI ≥ 5.6 resulted in a median survival time between 12 and 13 months. Tokyo score clarified the prognosis trend with smaller chi-square = 36.4 (*P* < .0001) than that in mMDN/ALBI and mUS/ALBI (Figure 4C).

**Figure 4.**
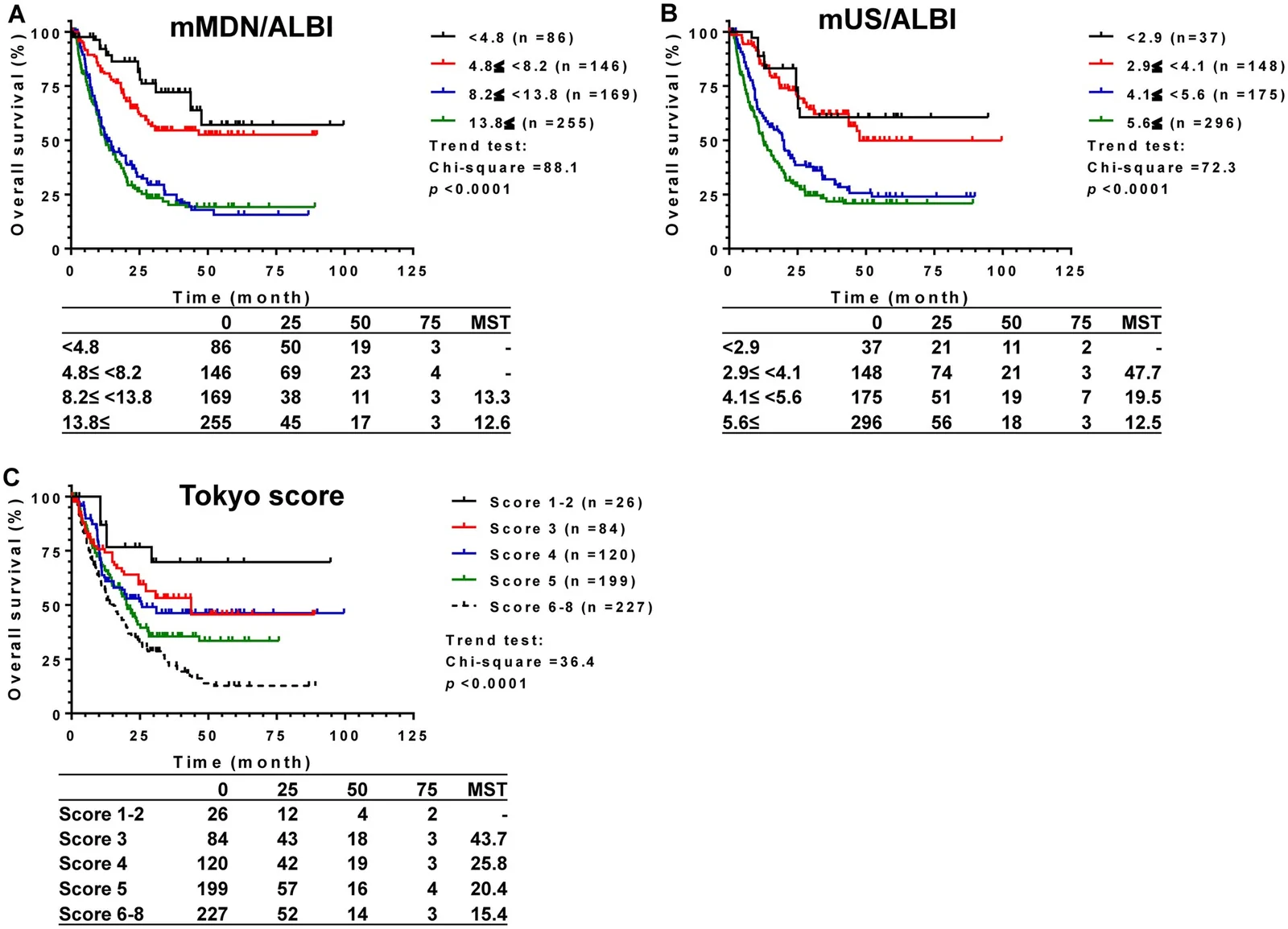
Stratification of overall survival by mMDN/ALBI and mUS/ALBI under the condition of the modified ALBI grade = 2b or 3. Prognosis prediction using novel indexes was compared to that using Tokyo score in patients with impaired functional hepatic reserve. Patients with the modified ALBI grade = 2b or 3 were selected to the analysis (*n* = 656). The cutoff values of mMDN/ALBI (4.8, 8.2, and 13.8) and mUS/ALBI (2.9, 4.1, and 5.6) were adopted from the quartile values of them in the total cohort. (A, B) mMDN/ALBI and mUS/ALBI stratified the overall survival of the patients with the modified ALBI grade 2b to 3 (chi-square = 88.1, *P* < .0001 for mMDN/ALBI; and chi-square = 72.3, *P* < .0001 for mUS/ALBI). mMDN/ALBI ≥ 8.2 suggested a poor median survival of 12 to 13 months. mUS/ALBI ≥ 5.6 also showed a median survival time of 12 months. (C) Chi-square value = 36.4 (*P* < .0001) for Tokyo score was smaller than that of mMDN/ALBI and mUS/ALBI. Abbreviations: HR, hazard ratio; MST, median survival time.

## Discussion

The present study constructed novel prognosis indexes for overall survival of patients with BCLC stage B HCC, by integrating conventional indicators of functional hepatic reserve and tumor burden, that is, the ALBI score, the maximum tumor diameter and the number of tumor nodules. Two novel indexes, mMDN/ALBI and mUS/ALBI, significantly stratified the overall survival of the cohort with more accuracy than that of conventional prognostic indexes. The superiority of 2 indexes were maintained in both cases of greater and smaller tumor burden. mMDN/ALBI ≥ 8.2 resulted in the shorter median survival time of around 12 to 14 months in both cases of high and low functional hepatic reserve. mUS/ALBI ≥ 5.6 was indicative of a median survival time of around 9 to 12 months, regardless of the functional hepatic reserve.

BCLC staging system for HCC was proposed by Llovet et al. in 1999.^5^ The system has been widely used as one of the standard HCC stage classifications internationally since then.^20^^,^^21^ BCLC classification incorporates multidimensional factors including tumor burden (size, number, vascular invasion, distant metastasis), functional hepatic reserve (Child–Pugh classification), performance status, and presence of symptoms. The most important feature is that the standard treatment policy according to the stage of the disease is described alongside.

The 2022 BCLC revision further incorporates more flexible treatment options and stratification including subclassification of BCLC stage B into stage B1 to B4.^7^ As BCLC stage B has traditionally been more heterogeneous, the number and size of tumors and functional hepatic reserve were considered to be the most important factors. Corresponding to the heterogeneous clinical manifestation in BCLC stage B, the revised classification has presented therapeutic options including liver resection, transplantation, and systemic therapy in addition to TACE, the conventional standard selection.

The novel prognosis indexes for patients with BCLC stage B HCC integrates the ALBI score and tumor burden. As the inputs of the novel indexes are continuous numbers, a wide range of functional hepatic reserve and tumor burden are acceptable. The outputs of the novel indexes are also continuous numbers. Thus, their interpretation is easy and cutoff values may be determined flexibly depending on clinical context.

The main advantage of the ratio-based model is its structural simplicity and interpretability, as it directly represents the balance between tumor burden and hepatic reserve. Because it does not require parameter estimation, the model is robust and less prone to overfitting, particularly in small datasets. In addition, the fixed functional form enhances generalizability across different cohorts, as it avoids sample-dependent weighting of covariates. Finally, the ratio formulation allows straightforward clinical interpretation and facilitates bedside applicability without computational requirements.

The novel prognosis indexes have several key strengths. First, they use objective indicators for functional hepatic reserve and tumor burden, such as, serum albumin, total bilirubin, the maximum tumor diameter, and number of tumor nodules. Second, they are independent of TNM classification, which can vary across academic conference and version updates,^18^^,^^22–24^ making them broadly applicable and consistent. Finally, these indexes describe tumor burden with continuous numbers rather than categories. This is a crucial advantage because TNM staging of HCC categorizes and oversimplifies the heterogeneity of tumor burden in patients with BCLC stage B HCC. For example, in the current cohort, the Japanese TNM staging system classified 97.5% of patients as stage 3, and the UICC-defined TNM classification similarly indicated stage 3 for all patients. Consequently, the JIS score and the mALBI-T score failed to stratify the spectrum of tumor burden within BCLC stage B. By using the maximum tumor diameter and number of tumor nodules as parameters, mMDN/ALBI and mUS/ALBI can quantify and rank the tumor burden of patients with BCLC stage B HCC in greater details.

In practice, mMDN/ALBI ≥ 8.2 may be a red flag in patients with BCLC stage B HCC in both cases of the mALBI grade 1 to 2a and 2b to 3. This value was associated with a suggested median survival time of 12 months to 14 months. Using mUS/ALBI, Similarly, mUS/ALBI ≥ 5.6 may also suggest a median survival time of 9 to 12 months regardless of the mALBI grade.

Conventionally, integral indexes of functional hepatic reserve and tumor burden, such as the JIS score and the mALBI-T score, have been reported from Japan.^11^^,^^13^^,^^14^ The JIS score combines the Child–Pugh classification with HCC staging based on JSH guidelines. The mALBI-T score integrates the mALBI grade and HCC staging defined by the JSH.^18^ As JIS and the mALBI-T score adopt the TNM classification for tumor burden, they have the advantage of distinguishing prognosis among a wide range of patients with TNM stage 1 to 4 HCC. However, reliance on TNM classification limits their ability to stratify prognoses specific to patients with BCLC stage B HCC. Therefore, patients with BCLC stage B HCC need prognosis indexes to measure tumor burden other than TNM systems.

Tokyo score describes tumor burden based on the maximum tumor diameter and number of tumor nodules.^14^ Tokyo score counts tumor number even in the case that tumor nodules are 5 or more in a liver. So, it might be said that Tokyo score more accurately quantifies tumor burden compared to mMDN/ALBI and mUS/ALBI. The weakness of Tokyo score may lie in the process to incorporate functional hepatic reserve into its formula. Tokyo score categorizes serum albumin and total bilirubin into 3 divisions, similar to Child–Pugh classification. But a past study revealed that the ALBI grade was superior to differentiating patients’ prognosis with Child A or B cirrhosis.^25^

While the current study did not cover it, BALAD2 score is another index of prognosis in patient with HCC, constructed by functional hepatic reserve and tumor factors.^26^ BALAD2 score totally consists of serum parameters including serum albumin, total bilirubin, AFP, DCP, and AFP-L3. Originally, these serum parameters were adopted into calculation as cutoff values in BALAD score.^27^ In BALAD2 score, the serum parameters were continuous variables. It should be noted that BALAD2 score was established based on wide range of patients with the TNM stage of HCC from 1 to 4, and BCLC stage from 0 to C, similar to the establishment of the ALBI grade.^26^ In contrast to BALAD2 and the ALBI grade, mMDN/ALBI and mUS/ALBI are specific to patients with BCLC stage B. In the current cohort, BALAD2 score was not evaluated for the prognostic ability because the current cohort lacked data of DCP or AFP-L3. AFP was not an independent risk factor in the current cohort partially because the patients were limited in BCLC stage B HCC.

This study has several limitations. First, the study population was limited to ethnicities in East Asia. Second, the present cohort lacks follow-up information about the therapeutic response to the initial TACE and subsequent treatments. Third, the etiology of underlying liver disease (eg, HBV, HCV, alcohol-related liver disease, and NASH) was not available even though disease etiology may substantially affect prognosis and treatment strategy. Fourth, immune checkpoint inhibitors were not used as a therapeutic option for patients in the cohort. Finally, the findings require further validation through additional studies to confirm their generalizability to other populations and treatment settings.

## Conclusion

The novel prognosis indexes specific to patients with BCLC stage B HCC stratified the overall survival of the patients based on both functional hepatic reserve and tumor burden. mMDN/ALBI ≥ 8.2 and mUS/ALBI ≥ 5.6 suggested that the median survival time was around 1 year.

## Supplementary Material

oyag392_Supplementary_Data

## Data Availability

The data analyzed in this study are available at https://datadryad.org/stash/dataset/doi:10.5061/dryad.pd44k8r.
