# Supplementary material for: New prognosis indexes specific to Barcelona Clinic Liver Cancer stage B hepatocellular carcinoma combining functional hepatic reserve and tumor burden
Source: Oncologist. 2026 Oct 5;31(11):oyag392. doi: 10.1093/oncolo/oyag392 (PMC13693367; doi:10.1093/oncolo/oyag392)
Supplement: Supplementary file 1 [file oyag392_supplementary_data.zip › Supplementary_Legends_2026.10.20.pdf]

Supplementary Figure 1. Flow chart of patient selection.

Among the total of 2,020 patients with BCLC stage B HCC, three patients with missing data on tumor location, 44 with ascites, insufficient documentation regarding the presence of ascites, or distant metastasis, and 28 with missing data on serum albumin or total bilirubin levels, and two with inappropriate description on serum albumin or total bilirubin, were excluded. Consequently, 1,843 patients were enrolled for further analysis.

Supplementary Figure 2. Correlation of novel prognosis indexes with functional hepatic reserve and tumor burden.

A) The ALBI score had a marginal correlation with tumor burden described by mMDN ($r = 0.0767$, $p = 0.0010$). B-C) A novel prognosis index, mMDN/ALBI, presented a strong correlation with mMDN ($r = 0.9428$, $p<0.0001$), and a weak correlation with the ALBI score ($r = 0.3783$, $p<0.0001$). D) The ALBI score had a negligible correlation with mUS, another tumor burden index ($r = 0.0642$, $p = 0.0059$). E-F) mUS/ALBI, another novel prognosis index, showed a strong correlation with mUS ($r = 0.8540$, $p<0.0001$), and a moderate correlation with the ALBI score ($r = 0.5402$,

p<0.0001).

95% CI, 95% confidence interval.

Supplementary Figure 3. Decision curve analysis

Decision curve analysis revealed differences in clinical utility among the evaluated indexes. The mMDN/ALBI-based model and mUS/ALBI-based model demonstrated greater net benefit compared to other models, outperforming both the "treat everyone" and "treat no one" strategies within a specific range of threshold probabilities.

Supplementary Figure 4. Stratification of overall survival based on tumor burden.

A-B) By using the median value of mMDN =20, the total cohort was divided into two sub-cohorts; one with higher tumor burden (n =935) and the other with the lower (n =908). Each sub-cohort was divided into four quartiles (Q1, Q2, Q3, and Q4) based on mMDN/ALBI in each sub-cohort. A) mMDN/ALBI significantly stratified the overall survival under condition that tumor burden was mMDN≥20 (chi-square =24.4, p<0.0001). A hazard ratio of Q4 over Q1 was 1.758 (95% CI: 1.439–2.292, p<0.0001). B) In the other sub-cohort with tumor burden of mMDN<20, mMDN/ALBI also showed a significant trend in the overall survival (chi-square =36.9, p<0.0001). A hazard ratio of Q4 over Q1 resulted in 2.423 (95% CI: 1.882–3.330, p<0.0001). C-D) In case of mUS/ALBI, the total cohort was split in half based on the tumor burden index using the median mUS =10 (n =967 in the mUS≥10 sub-cohort, and n =876 in the

mUS<10 sub-cohort). Each sub-cohort was divided into four quartiles (Q1, Q2, Q3, and Q4) based on mUS/ALBI in each sub-cohort. mUS/ALBI distinguished the overall survival of the sub-cohort with mUS≥10 (chi-square =24.7, p<0.0001) and other one with mUS<10 (chi-square =23.0, p<0.0001). Hazard ratios of Q4 over Q1 were HR =1.596 (95% CI: 1.299–2.031, p<0.0001) for the mUS≥10 sub-cohort, and HR =2.003 (95% CI: 1.513–2.719, p<0.0001) for the mUS<10 sub-cohort, respectively.

HR, hazard ratio; 95% CI, 85% confidence interval; MST, median survival time.
